# Flying High—Muscle-Specific Underreplication in *Drosophila*

**DOI:** 10.3390/genes11030246

**Published:** 2020-02-26

**Authors:** J. Spencer Johnston, Mary E. Zapalac, Carl E. Hjelmen

**Affiliations:** 1 Department of Entomology, Texas A&M University, College Station, TX 77843, USA; spencerj@tamu.edu; 2 Department of Biochemistry and Biophysics, Texas A&M University, College Station, TX 77843, USA; marebear@tamu.edu; 3 Department of Biology, Texas A&M University, College Station, TX 77843, USA

**Keywords:** endocycle, G0, G1, polyploidy, *Drosophila virilis*, *Drosophila melanogaster*, indirect flight muscle, flow cytometry

## Abstract

*Drosophila* underreplicate the DNA of thoracic nuclei, stalling during S phase at a point that is proportional to the total genome size in each species. In polytene tissues, such as the *Drosophila* salivary glands, all of the nuclei initiate multiple rounds of DNA synthesis and underreplicate. Yet, only half of the nuclei isolated from the thorax stall; the other half do not initiate S phase. Our question was, why half? To address this question, we use flow cytometry to compare underreplication phenotypes between thoracic tissues. When individual thoracic tissues are dissected and the proportion of stalled DNA synthesis is scored in each tissue type, we find that underreplication occurs in the indirect flight muscle, with the majority of underreplicated nuclei in the dorsal longitudinal muscles (DLM). Half of the DNA in the DLM nuclei stall at S phase between the unreplicated G0 and fully replicated G1. The dorsal ventral flight muscle provides the other source of underreplication, and yet, there, the replication stall point is earlier (less DNA replicated), and the endocycle is initiated. The differences in underreplication and ploidy in the indirect flight muscles provide a new tool to study heterochromatin, underreplication and endocycle control.

## 1. Introduction

While a species is known to have a basal ploidy level, with only the chromosome number from parents, individual tissue types and cells may differ in their copies of the genome or portions of the genome. Some tissues may become polyploid, in which every chromosome of the whole genome is copied within the cell [1]. This phenomenon may be referred to as endopolyploidy, while the process by which the genome is replicated without mitotic division is further referred to as endoreduplication, and cells which undergo this complete copying without division are often referred to as endocycling. There is, however, a unique instance in which replication is stalled before completion, referred to as underreplication. Underreplication (UR) occurs when DNA synthesis stalls during the synthesis (S phase) before completion of replication of heterochromatin and some late-replication euchromatin. The process is seen in select tissues in a wide variety of plants and animals [2]. Typically found in specialty tissues of Diptera, such as the well-studied polyploid salivary gland and ovarian follicle cells, UR results from a coupling of protein products from Rif1 and SuUR hindering progression of the DNA replication fork [3,4]. This stall usually occurs upon encountering heterochromatic DNA, which is tightly wound into nucleosomes, preventing this heterochromatic DNA from fully replicating [5,6]. While recent work has investigated the machinery involved in this stalling of replication, the mechanisms behind this process are not fully understood [2,7,8]. UR does not alter origin firing, or the initiation of replication [9], but rather determines how far the replication fork may progress during synthesis [10]. Estimations of the percent UR have been proposed to indicate the amount of heterochromatic DNA in the genome [11]. Comparisons across species shows that the proportion of underreplicated DNA is inversely correlated with genome size; the relative proportion of the genome replicated is less in larger genomes [11,12]. However, little work has investigated patterns of underreplication within a species, which may be further indicative of adaptive differences in heterochromatin, or possibly endoreduplication of specific euchromatic loci.

UR is typically accompanied by the endocycle, which produces UR polytene or polyploid nuclei [13]. In these cells, origin firing has been linked to the initiation of active gene transcription; cells that undergo polyploidy through the endocycle are large and highly metabolically active [9,10,14,15]. A variant of this process is seen in the thorax of *Drosophila*. Unlike UR in polytene cells, thoracic UR is seen as a stall of DNA synthesis between the G0 and G1 stage, with little evidence for endocycle initiation [16]. An exceptional property of thoracic UR involves the proportion of nuclei underreplicated. In tissues with polyploidy or polyteny, essentially all nuclei are underreplicated [17]. Yet, for unknown reasons, UR is seen in less than half the nuclei of the thorax [16]. If underreplication is adaptive, it would be expected that every cell would underreplicate. One possible explanation is that UR is only found in the nuclei of specific tissues of the thorax and not others. However, it is unknown whether half of the nuclei of all thoracic tissues are underreplicated, or if only a subset of tissues in the thorax are underreplicated, while others are not. If only specific tissues undergo UR, this information may be a clue towards understanding the adaptive nature of underreplication.

The thorax is comprised of a variety of different tissues, including cuticle, gut, tergal depressor of the trochanter (TDT) muscle, and the direct and indirect flight muscle [18]. Of these, only the gut is expected to have any polyploid nuclei, according to data collected from a bee and an ant [19,20]. Yet, gut nuclei are expected to be rare relative to the nuclei from other thoracic tissues, as the thorax is filled by muscle [21]. This thought is supported in this study by low numbers of cells at 8C and 16 in whole thoracic preparations; however, separate gut dissections and preparations were not performed. *Drosophila* thoracic muscles are comprised of direct flight muscles (DFMs), indirect flight muscles (IFMs), and the TDT muscles. Each of these muscle types have different fiber types. The fibers of the flight muscles and TDT are fibrillar and tubular, respectively [18]. IFMs are the largest group of muscles in the *Drosophila*. These IFMs can be further subdivided into dorsal longitudinal muscle (DLM) and dorsal ventral muscle (DVM), which have different functions [22,23] and different developmental pathways [21,24]. Three larval mesothoracic muscle fibers survive the wave of histolysis that destroys most of the larval body-wall musculature. These muscle fibers (MFs 9, 10 and 19) serve as scaffolds for the development of the six DLM fibers. In contrast, the DVM develops de novo from wing disk-derived myocytes. These differences in development and function have the potential to result in differing UR phenotypes between tissue types.

In order to address the question of “Why are nearly half the nuclei underreplicated in the thorax of *Drosophila*?”, UR was treated as a phenotype. In order to account for potential effects of different genome sizes, knowing that the thoracic UR stall point is inversely related to the amount of genomic DNA [11,12], we scored this phenotype in *Drosophila melanogaster* with its relatively small genome (1C = 175 Mbp) and in *Drosophila virilis* which has a relatively large genome (1C = 328 Mbp). We find that UR in the thorax occurs primarily, if not exclusively, in the indirect flight muscles of both species, with significant differences between the DLM and DVM. The major UR peak observed in whole thorax preparations is primarily from DLM nuclei, while the large G1 peak in those same preparations are primarily from DVM nuclei. The percent of DNA replicated up to the UR stall point in the DVM is half that in the DLM. The endocycle typical of underreplicated polytene tissues is initiated only in the DVM, and not in the DLM. The implications for the study of UR, the endocycle, and chromatin architecture are discussed.

## 2. Materials and Methods 

### 2.1. Dissection

*D. melanogaster* w1118] and the sequenced y,w from the Bloomington Stock Center and a lab strain of *D. virilis* maintained at the Johnston Lab at Texas A&M University were reared at room temperature, and prior to dissection, were frozen for at least 20 min at −80 °C. To aid tissue preparation and visualization, and reduce potential sources of variation, only 2–4-day-old females were prepared. Flies were briefly bathed in 70% ethanol to remove the wax layer of the cuticle and prevent floating in the buffer during dissection [25]. After washing with ethanol, each fly was submerged in Galbraith’s buffer (45 mM MgCl_2_, 30 mM sodium citrate, 20 mM MOPS, 0.10% v/v Triton X, pH 7.0) and the head, abdomen, wings, and legs were all removed. The cuticle was stripped away from the thorax to reveal the muscles. Muscles were located and identified according to Figure 5 of Dobi, et al. [26].

### 2.2. Measuring UR and Ploidy by Flow Cytometry

Nuclei from each muscle type for each strain were recovered for flow cytometry as described in Johnston et al. 2013 and scored for ploidy according to procedures in Scholes et al. (2014). To summarize, tissues were submerged in 1 mL of Galbraith buffer in a 2 mL Kontes Dounce homogenizer and ground gently with a maximum of 7 strokes. Contents were then filtered through 45 µM nylon mesh into a 1.5 mL Eppendorf tube. In total, 25 µL of propidium iodine (PI) at 1 mg/mL was added to each solution and vortexed for 10 s to mix. Each solution was incubated with stain for at least 20 min in the dark at 4 °C. Solutions were then run through a CytoFLEX flow cytometer (Beckman Coulter) with a blue laser emitting at 488 nM in order to measure fluorescence, and therefore relative DNA content of each nuclei. There were no discernable differences between y,w and w1118 strains; parameter values for *D. melanogaster* include results from both. 

### 2.3. Statistical Analysis

The cytometer scores total count and the mean fluorescence for each peak produced by the stained nuclei isolated from each tissue. As the amount of fluorescence measured may differ between samples based on variable such as PI staining intensity, flow rate, startup voltage, and voltage settings, all parameters are estimated within each sample based on the relative positions of the 2C, UR, and 4C peak before comparison between samples. This means that the position of each peak may vary between samples, but the relative positions of peaks within each sample will not differ, i.e., the 4C peak is always at a position with double the fluorescence as the 2C. This linearity in output allows calculations of proportion ploidy, underreplication, etc., which may be compared between samples. These data were summarized as five parameters: stall point, ploidy level, proportion in G0 (2C), proportion stalled, and proportion in G1 (4C).

The stall point was calculated by:

$$Stall\;point=\frac{UR\;peak\;position-2C\;peak\;position}{2C\;peak\;position}$$

Polyploidy was calculated as the average endoreduplication cycles per nucleus in each sample. These values were calculated as in Barow and Meister [27] using the following equation:

$$Ploidy=\frac{\left(0\times n2C\right)+\left(1\times n4C\right)+\left(2\times n8C\right)+\left(3\times n16C\right)\ldots }{n2C+n4C+n8C+n16C\ldots }$$

The ploidy level is 0.0 for unreplicated diploid cells at G0, 1.0 for fully replicated G1 cells, and increases incrementally with each successive round of endoreduplication. UR peaks are incorporated into the above as ((nUR/n2C)−1) × nUR, with nUR included in the denominator.

The proportion in G0, stall point, and G1 was calculated as the count for each peak divided by the total count for all peaks. The correct identification of the 2C peak was verified by regularly preparing a histogram from individual heads of dissected flies. The vast majority (90% or more) of nuclei from the head are 2C. 

Significance of mean differences among tissues for each parameter was tested using a Sheffe’s test with the means and type 3 error from a general linear ANOVA model, Y_ij_ = *µ* + Species_i_ + Tissue_j_ + Species*Tissue_ij_ + e*_ijk_*. 

A Principal Component Analysis was performed using all 5 parameters for each tissue using the function prcomp() in R.3.6.2 [28]. 

## 3. Results

### 3.1. Thoracic Underreplication Is a Property of Indirect Flight Muscle

**Figure 1:**
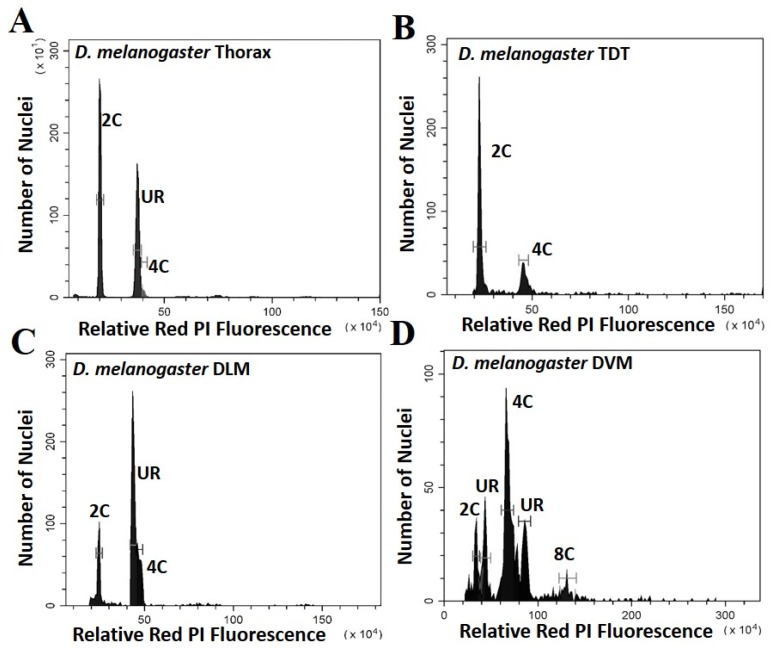
Histograms showing the peaks of fluorescence whose relative fluorescence is that expected of G0 (2C) and G1 (4C) nuclei for *Drosophila melanogaster*. Peaks at other relative positions represent underreplication and are labelled UR: (**A**) Whole thoracic preparations (**B**) Preparations of the tergal depressor of the trochanter (TDT) muscle; (**C**) Preparations of dorsal longitudinal indirect flight muscle (DLM); (**D**) Preparations of dorsal ventral flight muscle (DVM).

**Figure 2:**
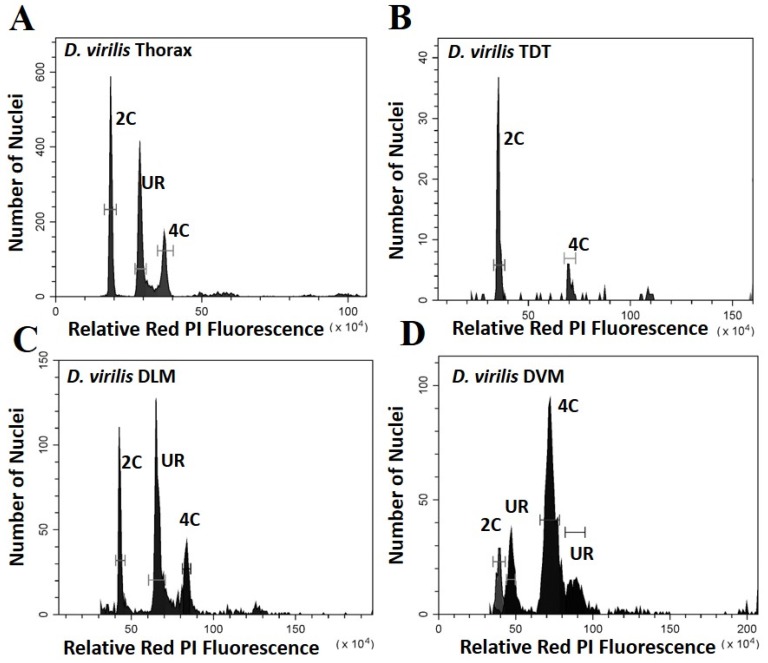
Histograms showing the peaks of fluorescence whose relative fluorescence is that expected of G0 (2C) and G1 (4C) nuclei for *Drosophila virilis*. Peaks at other relative positions represent underreplication and are labelled UR: (**A**) Whole thoracic preparations (**B**) Preparations of the tergal depressor of the trochanter (TDT) muscle; (**C**) Preparations of dorsal longitudinal indirect flight muscle (DLM); (**D**) Preparations of dorsal ventral flight muscle (DVM).

**Figure 3:**
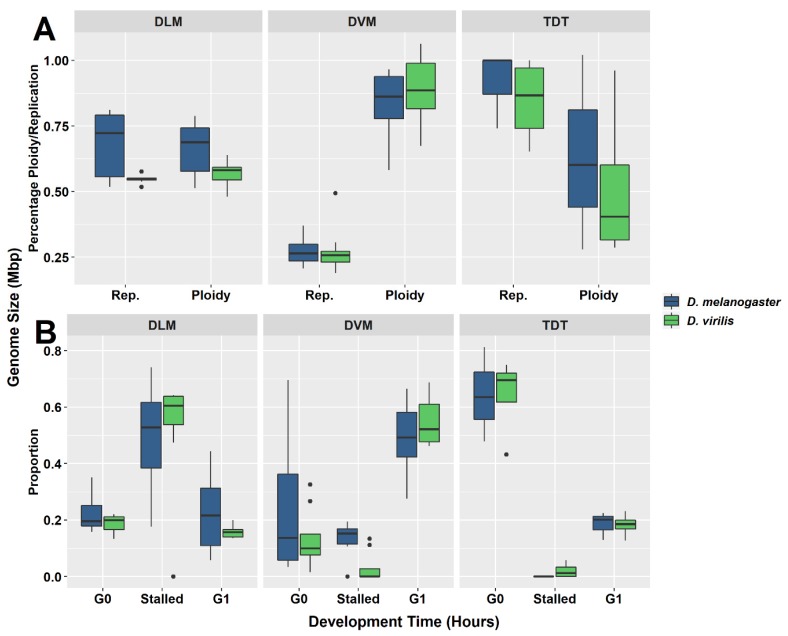
Box-and-whisker plots for each muscle type investigated in *D. melanogaster* and *D. virilis*. (**A**) Distribution of ploidy percentage and replication percentage across tissues. (**B**) Distribution of proportion of nuclei at G0, G1, or stalled in replication in each muscle type. Each muscle type presents a unique pattern. The intermediate levels of ploidy and replication in dorsal longitudinal muscle (DLM) tissues (**A**) represent the largest proportion of nuclei stalled during replication (**B**). The high ploidy percentage ploidy in the dorsal ventral muscle (DVM) (**A**) is due to the high proportion of nuclei found at the G1 phase (**B**). Almost no nuclei are found to be stalled during replication in the tergal depressor of the trochanter (TDT) muscle and are predominately in the G0 phase (**B**).

Five parameters of the cell cycle were scored from thoraxes and muscles dissected from multiple individuals from each species, as seen by representative histograms in Figure 1 and Figure 2. Additional peaks beyond 4C are often observed (as in Figure 1D) and may represent additional stall points. All peaks are scored and included in calculation of ploidy parameters. However, because these “other” peaks have few nuclei, they have very minor effects on the values. The only stall points that are large, common to all replicates, and have significant effects on the parameters are those labelled in Figure 1 and Figure 2. These parameters (proportion G0, G1, stalled replication, percentage replication and percentage ploidy) are summarized in Table 1 and in Figure 3. These show that the vast majority of UR seen in whole thorax preparations is from the nuclei of DLM, where approximately half of the nuclei are underreplicated. UR in the DLM (the large peak seen in Figure 1C and Figure 2C is 68% above the G0 peak in *D. melanogaster*, and at 55% above the G0 peak in *D. virilis*. In addition, the DLM have a limited number of nuclei at G0 (19% of scored nuclei in *D. melanogaster* and 22% in *D. virilis*) and another relatively small proportion of nuclei fully replicated in G1 (16% in *D. melanogaster* and 24% in *D. virilis*). There is little or no endocycle in the DLM, and essentially no nuclei at higher ploidy levels.

The DVM shows a very different underreplication and ploidy profile compared to the DLM (Figure 1C,D, Figure 2C,D and Figure 3). Approximately half the nuclei in the DVM are fully replicated at G1 (55% in *D. melanogaster* and 49% in *D. virilis*). Replication stalls between G0 and G1 significantly earlier in the DVM in both species (13% in *D. melanogaster* and 15% in *D. virilis*), with a second UR peak above the G1. This latter reflects initiation of the endocycle, with the endopolyploid UR peak at the same percentage (15%) above the G0 and G1 in both species. The other muscles of the thorax are as represented by the TDT (Figure 1B and Figure 2B) and direct flight muscles (not shown) have nuclei that are almost entirely unreplicated G0 (64% in *D. melanogaster* and *D. virilis*) with a minor peak of fully replicated nuclei at G1 (18–19% in *D. melanogaster* and *D. virilis*). The TDT muscles show no evidence of an endocycle, with few, if any, polyploid nuclei, and very few instances of underreplicated nuclei (Table 1, Figure 1B and Figure 2B).

### 3.2. Standard Errors and Individual Tissue Points Plotted in a Principal Comonent Analysis Show Variation within and among the Tissue Dissected from Different Individuals

**Figure 4:**
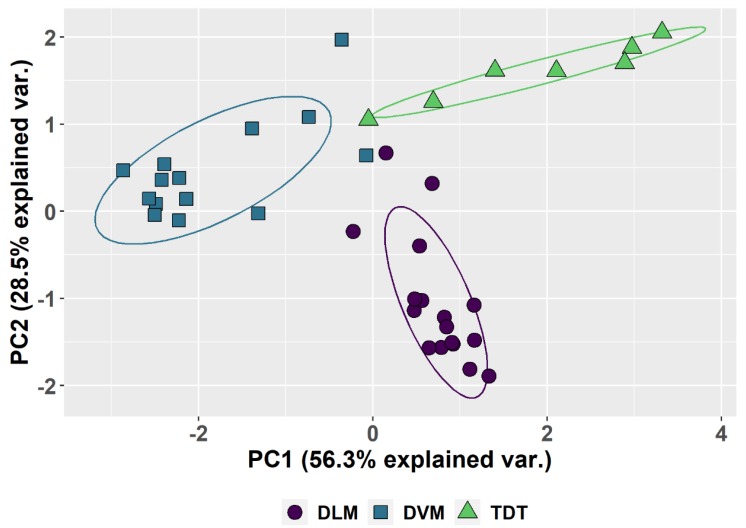
Principal Component Analysis based on the five parameters in Table 1. PCA 1 and PCA 2 are calculated and plotted for all the muscles scored. The differences between the dorsal longitudinal muscles (DLM), the dorsal ventral groups (DVM) and tergal depressor of the trochanter (TDT) muscles are the major source of variation and circled to show the common features of each. The variation between different muscles when dissected from different individuals produces the observed spread. Species differences are not shown and not the source of variation.

Although there is considerable individual variation in the parameters (Table 1, Figure 3), a common pattern is observed for each tissue type (Figure 3A,B). The patterns and the variation among individuals are plotted in box-and-whisker format (Figure 3) as well as in the PCA (Figure 4). The first two principal components account for 85% of the total variation. Component 1 accounts for 56% of the variation and reflects a pattern that consists of 0.51 of replication stall point, 0.417 of the proportion of G0, −0.500 ploidy and −0.526 of the proportion in G1 with little input (0.191) from the proportion replicated (UR). Component 2 accounts for an additional 29% of the variation and reflects a second pattern consisting of −0.775 of the proportion replication and 0.6 of the proportion in G0, with minimal input from the other parameters. Circles drawn about the different tissue types show the common patterns shared by DVM, DLM and TDT.

## 4. Discussion

Thoracic UR is limited to two muscle types, the dorsal ventral (DVM) and dorsal longitudinal (DLM) indirect flight muscle. Both of these have a mix of G0, underreplicated, and G1 nuclei. The DLM contributes most of the nuclei scored in thoracic preparations and has the largest proportion of UR nuclei (Table 1 and Figure 3). The replication profile seen for DVM is largely hidden by background variation in whole thorax preparations, and except for the DVM contribution to the G1 peak, the DVM profile can be missed in whole thorax preparations (Figure 1 and Figure 2). The replication profiles of the DLM and DVM are very different when prepared individually (Figure 1, Figure 2 and Figure 3). In the DVM, the stall point is earlier than that of the DLM and there is initiation of the endocycle. The large proportion of G1 nuclei and an endopolyploid replication peak that appears above the G1 in the DVM results in a higher ploidy level than in the DLM (Table 1, Figure 3). While UR in the DLM proceeds further, it stops between G0 and G1, independent of the endocycle. No other tissue is known to underreplicate without initiating the endocycle, replicating beyond G1.

The different ploidy level of the DLM and DVM, and possibly the earlier stall point in the DVM, may be explained by the different developmental pathway of the two muscle types [29,30]. The process is, however, counterintuitive. The DVM develops de novo during pupation from myocytes derived from the wing disk that are presumably diploid [21,24]. Those same progenitor cells are undifferentiated out of necessity and may have primarily centromeric heterochromatin and little facultative heterochromatin [31]. What we see, however, is an early stall of replication. The DLM is organized from larval muscle, and the DLM might be expected to reflect the larval condition. That is, DLM is organized from larval tissues that are fully differentiated, often polyploid, with more facultative heterochromatic DNA; yet, the proportion replicated is greater in the DLM. The earlier occurrence of the replication stall point suggests increased proportions of heterochromatin or endoreduplication at specific euchromatic loci. However, to fully identify this, copy number information and identification of euchromatic and heterochromatic sequences must be gleaned from tissue specific sequencing efforts. Differences in heterochromatization or endoreduplication at specific loci suggests that function rather than development may be responsible for the UR and ploidy differences. However, this conjecture is lacking experimental evidence.

The different tissues of the thorax provide an opportunity to study underreplication either with or without initiation of the endocycle. The pattern of underreplication in the DLM mimics the pattern seen in preparation of the whole thorax (Figure 1A,C and Figure 2A,C) with a strong G0 peak, an even larger peak at the replication stall point (UR), and a relatively small G1 peak. The DLM nuclei produce the replication stall point observed in whole thoracic preparations, which is thought to reflect the proportion of euchromatic DNA, and exhibits an inverse correlation with genome size [11]. Uniquely, the DLM has essentially no nuclei with replication beyond the G1, and consequently no evidence for the endocycle. The DVM also underreplicates, but the proportion of total DNA replicated is reduced by half, and underreplication appears twice, the first above the G0 and the second above the G1. These differences in patterns here suggest that UR may reflect adaptive chromatin landscapes that may be specific to provide unique support to each of these muscle types.

Observed variation within tissues in the controlled laboratory environment here may be exacerbated in wild-type populations where differences may reflect environment effects and somatic variation. In *Phaseolus vulgaris* (the common bean) reared at lower temperatures there is increased visibility of polytene chromosome bands, which results in more compact polytene chromosomes; while at higher temperatures, there is a reduction in banding during growth [32]. A correlated change in phenotype of underreplication may occur in *Drosophila* and explain why cactophilic *Drosophila* in the *nannoptera* group, that experience extreme heat, have exceptional amounts of heterochromatin [33]. Somatic heterogeneity is also a likely source of variation. Somatic variation in underreplication is well documented for polytene salivary glands [34], while stress induces polyploidy and cell fusion [13,35]. Stress has been documented to result in epigenetic changes in the chromatin landscape [36]. Additionally, it was found that outside stimuli can regulate dynamic changes in constitutive heterochromatin [37].

The most curious observation is the early stall point in the DVM. Synthesis during a normal cell cycle is known to stall when the replication fork encounters heterochromatin. This has been taken to mean that the proportion of euchromatin can be estimated from the stall point in polytene nuclei [11]. The same might be expected of the stall point in the thoracic nuclei. Yet, while the relationship of the stall point to heterochromatin may hold true for the DLM and be reflected in the whole thorax preparations, it cannot be true of the nuclei of DVM, because of the earlier stall point in replication. This difference complicates the assignment of partial replication to a phenotype. It remains to be seen if the DNA is more compact in the DVM, with a larger percentage of heterochromatin, or if specific loci are endoreduplicated. The latter is anticipated in the work of Windner, et al. [38] who show observed variation in size among the many nuclei in single muscle cells. They mention that this size variation likely reflects the amount and proportion of underreplication and the ploidy level in individual nuclei, but do not compare variation in DVM and DLM.

Much remains to be studied with regard to the phenotype of underreplication in the thorax of *Drosophila*. Although the tissues show highly significant mean differences in the measured parameters, there is nonetheless considerable variation between individuals (Figure 4). Whether parameters change with age remains to be quantified. Also to be determined are environmental effects on the parameters. Flight performance in honeybees is associated with a reduction in polyploidy with age [19]. Further, while it is known that endocyclic cells are large and metabolically active, have multiple sites of origin firing, and variable levels of transcription [10,14,15], it remains to be seen whether underreplication is a mechanism to meet the metabolic demands of flight with a minimal increase in nuclear size. The identification of differing patterns of underreplication in indirect flight muscle tissues opens an interesting resource to pursue questions of underreplication, development, and tissue specific adaptation. 

## Figures and Tables

**Table 1:** Mean replication parameters with standard errors in parentheses. Stall point is the proportion of DNA replicated between G0 and G1. Ploidy is as described in methods. The proportions of nuclei in G0, partial replication (stalled) and G1 peaks is relative to the total nuclei scored for that tissue. The means are shown by species. However, a Scheffe test found no significant species by tissue interactions and no significant parameter mean differences between species. Bold and underlined letters represent unique, highly significant mean parameter Scheffe test differences across tissues.

	Muscle Type			
***D. melanogaster***	**TDT**	**DLM**	**DVM**	**Significance**
N	4	8	8	
Average Nuclei Count	721	1017	392	
Stall point	0.85 (0.08) **A**	0.55 (0.01) **B**	0.13 (0.03) **C**	*p* < 0.0001
Ploidy level	0.51 (0.15) A	0.57 (0.02) A	0.89 (0.05) **B**	*p* < 0.0005
Proportion in G0	0.64 (0.07) **A**	0.19 (0.01) B	0.13 (0.04) B	*p* < 0.0001
Proportion stalled	0.02 (0.01) A	0.52 (0.08) **B**	0.03 (0.02) A	*p* < 0.0001
Proportion in G1	0.18 (0.02) A	0.16 (0.01) A	0.55 (0.03) **B**	*p* < 0.0001
***D. virilis***	**TDT**	**DLM**	**DVM**	
N	3	9	6	
Average Nuclei Count	442	1188	803	
Stall point	0.91 (0.09) **A**	0.68 (0.04) **B**	0.15 (0.02) **C**	*p* < 0.0001
Ploidy level	0.63 (0.21) A	0.66 (0.03) A	0.83 (0.06) **B**	*p* < 0.0005
Proportion in G0	0.64 (0.10) **A**	0.22 (0.02) B	0.25 (0.11) B	*p* < 0.0001
Proportion stalled	0.00 (0.00) A	0.51 (0.06) **B**	0.13 (0.03) A	*p* < 0.0001
Proportion in G1	0.19 (0.03) A	0.24 (0.05) A	0.49 (0.06) **B**	*p* < 0.0001
